# Supplementary material for: Empirical methods for controlling false positives and estimating confidence in ChIP-Seq peaks
Source: BMC Bioinformatics. 2008 Dec 5;9:523. doi: 10.1186/1471-2105-9-523 (PMC2628906; doi:10.1186/1471-2105-9-523)
Supplement: Additional File 1 — A variety of html documents from the USeq web site detailing the available applications, their best usage, output file type descriptions, command line menus, etc. [file 1471-2105-9-523-S1.zip › USeqUserGuides/JavaDoc.zip!/JavaDoc/bioroot/strain/StrainBean.html]

StrainBean


|  |  |  |
| --- | --- | --- |
| **Overview** \| **Package** \| **Class** \| **Use** \| **Tree** \| **Deprecated** \| **Index** \| **Help** | |  |
| **PREV CLASS**   **NEXT CLASS** | **FRAMES**    **NO FRAMES**     **All Classes** |  |
| SUMMARY: NESTED \| FIELD \| CONSTR \| METHOD | DETAIL: FIELD \| CONSTR \| METHOD |  |


---


## bioroot.strain Class StrainBean

```
java.lang.Object
  bioroot.ReagentBean
      bioroot.strain.StrainBean
```

**All Implemented Interfaces:**: java.lang.Comparable

---

``` public class StrainBean extends ReagentBean ```

**Author:**
:   nix
    Container for holding Strain information

---

**Field Summary**

**Fields inherited from class bioroot.ReagentBean**

`log`


**Constructor Summary**

`StrainBean()`

`StrainBean(int id,
DBUtil bioRoot)`

`StrainBean(java.sql.ResultSet results)`

`StrainBean(java.lang.String[] cells,
IdNameMatch[] cellTypes,
IdNameMatch[] organisms,
IdNameMatch[] markerIDMatch,
UserBean userBean,
DBUtil bioRoot)`
  
          For bulk file upload should be 23 cells
0 Name
1 Alias
2 Source
4 Reference
5 Type Bacteria, Yeast, Worm, Insect, Plant, Fish, Cell Line, Bird, Mammal or Other
6 Recipients
7 Organism name
8 Markers names, comma seperated
9 Plasmid Ids
10 Parent Strain Ids
11 Storage Location
12 Mutagen EMS, ENU, MMS, MNU, X-ray, UV, Transposon, T-DNA, Spontaneous or Other
13 Genotype/ Background
14 Phenotype
15 Growth Conditions
16 Restock
17 Remaining Aliquots
18 Array Type Extra Chromosomal or Integration
19 Seed Type Individual Line, Individual Pool, Set of Lines, or Set of Pools
20 Cell Type (Cell Lines)
21 Passage Number ( Cell Lines)
22 Mating Type ( Yeast) diploid, a, alpha, h+, or h-)
23 Availability
24 VisibilityBean
25 Notes


| **Method Summary** | |
| --- | --- |
| `int` | `compareTo(java.lang.Object obj)` |
| `java.lang.String` | `getArrayType()` |
| `int` | `getCellTypeId()` |
| `java.lang.String` | `getCellTypeName(DBUtil bioRoot)`             Will look one time for a name, all other times will just return "". |
| `java.lang.String` | `getFileName()` |
| `java.lang.String` | `getGenotype()` |
| `java.lang.String` | `getGrowthConditions()` |
| `java.lang.String` | `getHotLinkedParents(DBUtil bioRoot)`             Returns HTML parent/ strain names for display. |
| `java.lang.String` | `getHotLinkedPlasmids(DBUtil bioRoot)`             Returns HTML plasmid names for display. |
| `java.lang.String[]` | `getMarkers(DBUtil bioRoot)` |
| `java.lang.String` | `getMatingType()` |
| `java.lang.String` | `getMutagen()` |
| `java.lang.String[]` | `getParentConcats(DBUtil bioRoot)`             Returns an array of id: strainName, or empty String[]{""} if there are none. |
| `java.lang.String` | `getPassageNumber()` |
| `java.lang.String` | `getPhenotype()` |
| `java.lang.String` | `getPlainTextParent()`             Returns plain text of parent ids for archive. |
| `java.lang.String` | `getPlainTextPlasmid()`             Returns plain text of plasmid ids for archive. |
| `java.lang.String[]` | `getPlasmidConcats(DBUtil bioRoot)`             Returns an array of id: plasmidName, or empty String[]{""} if there are none. |
| `java.lang.String` | `getRecipients()` |
| `java.lang.String` | `getRemainingAliquots()` |
| `java.lang.String` | `getRestock()` |
| `java.lang.String` | `getSeedType()` |
| `java.lang.String` | `getSource()` |
| `java.lang.String` | `getType()` |
| `boolean` | `isStrainNameUnique(DBUtil bioRoot)`             checks to see if strain name is unique within labgroup, assumes a connection has been made |
| `void` | `loadStrainBean(java.sql.ResultSet results)` |
| `boolean` | `saveMarkers(DBUtil bioRoot, boolean deleteOld)` |
| `boolean` | `saveParentIds(DBUtil bioRoot, boolean deleteOld)`             Can delete old strainIds from StrainParent table, and add new entries if any are present in the parentConcats. |
| `boolean` | `savePlasmidIds(DBUtil bioRoot, boolean deleteOld)`             Can delete old plasmidIds from StrainPlasmid table, and add new entries if any are present in the plasmidConcats. |
| `void` | `setArrayType(java.lang.String arrayType)` |
| `void` | `setCellTypeId(int tissueId)` |
| `void` | `setCellTypeName(java.lang.String tissueName)` |
| `void` | `setFileName(java.lang.String fileName)` |
| `void` | `setGenotype(java.lang.String genotype)` |
| `void` | `setGrowthConditions(java.lang.String growthConditions)` |
| `void` | `setMarkers(java.lang.String[] markers)` |
| `void` | `setMatingType(java.lang.String matingType)` |
| `void` | `setMutagen(java.lang.String mutagen)` |
| `void` | `setParentConcats(java.lang.String[] parentConcats)` |
| `void` | `setPassageNumber(java.lang.String passageNumber)` |
| `void` | `setPhenotype(java.lang.String phenotype)` |
| `void` | `setPlasmidConcats(java.lang.String[] plasmidConcats)` |
| `void` | `setRecipients(java.lang.String recipients)` |
| `void` | `setRemainingAliquots(java.lang.String remainingAliquots)` |
| `void` | `setRestock(java.lang.String restock)` |
| `void` | `setSeedType(java.lang.String seedType)` |
| `void` | `setSource(java.lang.String source)` |
| `void` | `setType(java.lang.String type)` |
| `boolean` | `submitNew(DBUtil bioRoot)`             Makes a new Strain table entry, don't forget to save associated markers and plasmids after updating this bean with its new id number. |
| `boolean` | `updateOld(DBUtil bioRoot, UserBean submitter)`             Updates a Strain entry, don't forget to update markers and plasmids. |
| `boolean` | `updateStrainCommonAccessFields(DBUtil bioRoot)`             Just updates fields that are accessible by all lab group memebers. |

**Methods inherited from class bioroot.ReagentBean**

`appendMessages, canModifyAll, canModifyCommon, canView, getAlias, getAvailability, getComments, getEditHistory, getGenericVisibility, getId, getLabGroupId, getLabGroupName, getLastUser, getLocation, getMessages, getName, getNotes, getOrderBy, getOrganismId, getOrganismName, getOrganization, getOwnerId, getOwnerName, getReference, getVisibility, isComplete, isReagentNameUnique, loadReagentBean, setAlias, setAvailability, setComments, setComplete, setEditHistory, setId, setLabGroupId, setLabGroupName, setLastUser, setLocation, setMessages, setName, setNotes, setOrderBy, setOrganismId, setOrganismName, setOwnerId, setOwnerName, setReference, setVisibility, updateCommonAccessFields`

**Methods inherited from class java.lang.Object**

`equals, getClass, hashCode, notify, notifyAll, toString, wait, wait, wait`

**Constructor Detail**

### StrainBean

```
public StrainBean()
```

---


### StrainBean

```
public StrainBean(int id,
                  DBUtil bioRoot)
```

---


### StrainBean

```
public StrainBean(java.sql.ResultSet results)
```

---


### StrainBean

```
public StrainBean(java.lang.String[] cells,
                  IdNameMatch[] cellTypes,
                  IdNameMatch[] organisms,
                  IdNameMatch[] markerIDMatch,
                  UserBean userBean,
                  DBUtil bioRoot)
```

:   For bulk file upload should be 23 cells
    0 Name
    1 Alias
    2 Source
    4 Reference
    5 Type Bacteria, Yeast, Worm, Insect, Plant, Fish, Cell Line, Bird, Mammal or Other
    6 Recipients
    7 Organism name
    8 Markers names, comma seperated
    9 Plasmid Ids
    10 Parent Strain Ids
    11 Storage Location
    12 Mutagen EMS, ENU, MMS, MNU, X-ray, UV, Transposon, T-DNA, Spontaneous or Other
    13 Genotype/ Background
    14 Phenotype
    15 Growth Conditions
    16 Restock
    17 Remaining Aliquots
    18 Array Type Extra Chromosomal or Integration
    19 Seed Type Individual Line, Individual Pool, Set of Lines, or Set of Pools
    20 Cell Type (Cell Lines)
    21 Passage Number ( Cell Lines)
    22 Mating Type ( Yeast) diploid, a, alpha, h+, or h-)
    23 Availability
    24 VisibilityBean
    25 Notes


**Method Detail**

### compareTo

```
public int compareTo(java.lang.Object obj)
```

:   **Specified by:**: `compareTo` in interface `java.lang.Comparable` **Overrides:**: `compareTo` in class `ReagentBean`

---


### getCellTypeName

```
public java.lang.String getCellTypeName(DBUtil bioRoot)
```

:   Will look one time for a name, all other times will just return "".

---


### getPlasmidConcats

```
public java.lang.String[] getPlasmidConcats(DBUtil bioRoot)
```

:   Returns an array of id: plasmidName, or empty String[]{""} if there are none.

---


### savePlasmidIds

```
public boolean savePlasmidIds(DBUtil bioRoot,
                              boolean deleteOld)
```

:   Can delete old plasmidIds from StrainPlasmid table, and add new entries if any are
    present in the plasmidConcats.

---


### getHotLinkedPlasmids

```
public java.lang.String getHotLinkedPlasmids(DBUtil bioRoot)
```

:   Returns HTML plasmid names for display.

---


### getPlainTextPlasmid

```
public java.lang.String getPlainTextPlasmid()
```

:   Returns plain text of plasmid ids for archive.

---


### getMarkers

```
public java.lang.String[] getMarkers(DBUtil bioRoot)
```

---


### setMarkers

```
public void setMarkers(java.lang.String[] markers)
```

---


### saveMarkers

```
public boolean saveMarkers(DBUtil bioRoot,
                           boolean deleteOld)
```

---


### getParentConcats

```
public java.lang.String[] getParentConcats(DBUtil bioRoot)
```

:   Returns an array of id: strainName, or empty String[]{""} if there are none.

---


### saveParentIds

```
public boolean saveParentIds(DBUtil bioRoot,
                             boolean deleteOld)
```

:   Can delete old strainIds from StrainParent table, and add new entries if any are
    present in the parentConcats.

---


### getHotLinkedParents

```
public java.lang.String getHotLinkedParents(DBUtil bioRoot)
```

:   Returns HTML parent/ strain names for display.

---


### getPlainTextParent

```
public java.lang.String getPlainTextParent()
```

:   Returns plain text of parent ids for archive.

---


### loadStrainBean

```
public void loadStrainBean(java.sql.ResultSet results)
```

---


### submitNew

```
public boolean submitNew(DBUtil bioRoot)
```

:   Makes a new Strain table entry, don't forget to save associated markers and plasmids after
    updating this bean with its new id number.

---


### updateOld

```
public boolean updateOld(DBUtil bioRoot,
                         UserBean submitter)
```

:   Updates a Strain entry, don't forget to update markers and plasmids.

---


### updateStrainCommonAccessFields

```
public boolean updateStrainCommonAccessFields(DBUtil bioRoot)
```

:   Just updates fields that are accessible by all lab group memebers.

---


### isStrainNameUnique

```
public boolean isStrainNameUnique(DBUtil bioRoot)
```

:   checks to see if strain name is unique within labgroup, assumes a connection has been made

---


### getFileName

```
public java.lang.String getFileName()
```

---


### setFileName

```
public void setFileName(java.lang.String fileName)
```

---


### getSource

```
public java.lang.String getSource()
```

---


### setSource

```
public void setSource(java.lang.String source)
```

---


### getGenotype

```
public java.lang.String getGenotype()
```

---


### setGenotype

```
public void setGenotype(java.lang.String genotype)
```

---


### getGrowthConditions

```
public java.lang.String getGrowthConditions()
```

---


### setGrowthConditions

```
public void setGrowthConditions(java.lang.String growthConditions)
```

---


### getRemainingAliquots

```
public java.lang.String getRemainingAliquots()
```

---


### setRemainingAliquots

```
public void setRemainingAliquots(java.lang.String remainingAliquots)
```

---


### getPassageNumber

```
public java.lang.String getPassageNumber()
```

---


### setPassageNumber

```
public void setPassageNumber(java.lang.String passageNumber)
```

---


### getPhenotype

```
public java.lang.String getPhenotype()
```

---


### setPhenotype

```
public void setPhenotype(java.lang.String phenotype)
```

---


### setPlasmidConcats

```
public void setPlasmidConcats(java.lang.String[] plasmidConcats)
```

---


### getRestock

```
public java.lang.String getRestock()
```

---


### setRestock

```
public void setRestock(java.lang.String restock)
```

---


### setCellTypeName

```
public void setCellTypeName(java.lang.String tissueName)
```

---


### getType

```
public java.lang.String getType()
```

---


### setType

```
public void setType(java.lang.String type)
```

---


### getCellTypeId

```
public int getCellTypeId()
```

---


### setCellTypeId

```
public void setCellTypeId(int tissueId)
```

---


### getArrayType

```
public java.lang.String getArrayType()
```

---


### setArrayType

```
public void setArrayType(java.lang.String arrayType)
```

---


### getMatingType

```
public java.lang.String getMatingType()
```

---


### setMatingType

```
public void setMatingType(java.lang.String matingType)
```

---


### getMutagen

```
public java.lang.String getMutagen()
```

---


### setMutagen

```
public void setMutagen(java.lang.String mutagen)
```

---


### getSeedType

```
public java.lang.String getSeedType()
```

---


### setSeedType

```
public void setSeedType(java.lang.String seedType)
```

---


### setParentConcats

```
public void setParentConcats(java.lang.String[] parentConcats)
```

---


### getRecipients

```
public java.lang.String getRecipients()
```

---


### setRecipients

```
public void setRecipients(java.lang.String recipients)
```


---


|  |  |  |
| --- | --- | --- |
| **Overview** \| **Package** \| **Class** \| **Use** \| **Tree** \| **Deprecated** \| **Index** \| **Help** | |  |
| **PREV CLASS**   **NEXT CLASS** | **FRAMES**    **NO FRAMES**     **All Classes** |  |
| SUMMARY: NESTED \| FIELD \| CONSTR \| METHOD | DETAIL: FIELD \| CONSTR \| METHOD |  |


---
